# Mobile health clinics in a rural setting: a cost analysis and time motion study of La Clínica in Oregon, United States

**DOI:** 10.1186/s12913-024-12203-5

**Published:** 2025-01-17

**Authors:** Abigail Higgins, Middy Tilghman, Tracy Kuo Lin

**Affiliations:** 1 Drexel University College of Medicine, 60 N. 36Th Street, Philadelphia, PA 19104 USA; 2 One Community Health, 849 Pacific Ave, Hood River, OR 97031 USA; 3 University of California, San Francisco Institute for Health & Aging, #123K, 490 Illinois Street, San Francisco, CA 94158 USA

**Keywords:** Mobile Health Clinic, Rural Health, Cost Analysis, Population Health, Time motion study, Resources utilization

## Abstract

**Background:**

Mobile Health Clinics (MHCs) are an alternate form of healthcare delivery that may ameliorate current rural–urban health disparities in chronic diseases and have downstream impacts on the health system by reducing costs. Evaluations of providers’ time allocation on MHCs are scarce, hindering knowledge transfer related to MHC implementation strategies.

**Methods:**

Retrospective economic cost was assessed using business ledgers and expert assessments in 2023 US Dollar (USD) from 2022 to 2023. Time motion observational study assessed nurse practitioner (NP) and community health worker (CHW) time allocation and compared them between patients residing in isolated rural areas (hereafter isolated rural patients) and patients experiencing houselessness (PEH) sub-populations. Procedure codes were assessed retrospectively for each patient encounter (*n* = 1,981) over one year (April 2022 to April 2023). We used statistical significance tests (chi-square and Fisher’s Exact) to evaluate difference across sub-populations.

**Results:**

Intervention start-up and operational costs totaled 275,000USD and 308,000USD, respectively, with the largest allocations to the modified recreational vehicle (RV) unit and labor. NP attributed 32% of time on direct care (mean = 153.00 min (SD = 37.80 min)), 38% on indirect care (186.0 (53.40)), and 21% on MHC tasks (104.00 (23.94)). CHW spent 47% of time on MHC tasks (182.00 (29.46)), 22% on medical care tasks (85.01 (SD 81.97)), and 22% on social needs tasks (87.70 (86.71 min)). NP time allocation did not differ significantly between isolated rural patients and PEH (*p* > 0.01), but CHW time did (*p* < 0.01). Of all procedures, 31.3% were vaccinations (*N* = 438), 27.0% were Covid-19 related (*N* = 377), 12.8% were outside referrals (*N* = 179), and 11.8% were point of care testing. Healthcare utilization varied between patient sub-populations, with Isolated Rural patient use dominated by Covid-19 and Influenza vaccines whereas PEH use was dominated by point of care testing (*p* < 0.01).

**Conclusion:**

Patient sub-populations require varying provider time in different tasks and variable economic resources for interventions. As local policy makers balance resources and community health needs, a complete understanding of the resources required to operate an MHC and use of provider time is essential for informed decision making and successful implementation in underserved communities.

**Supplementary Information:**

The online version contains supplementary material available at 10.1186/s12913-024-12203-5.

## Background

There exist significant health disparities between rural and urban populations in the United States (US) [1, 2]. The intersection of poorer access to healthcare, lower socioeconomic status, and higher rates of health risk behaviors, such as smoking and alcohol use, are considered drivers of disparities in rural health outcomes – referred to as “The Rural Mortality Penalty” [2–4]. The disproportionally poorer access to healthcare in rural communities are cited to be due to various social, economic, and systemic barriers that prevent seeking and receiving quality medical care [5, 6]. Social barriers such as poor health literacy and social stigma in doctor’s offices prevent rural community members from accessing care [6, 7]. Economic barriers, including a lack of health insurance and high out-of-pocket payments, are associated with patients in rural settings postponing or refraining from primary care health visits [7]. Systemic barriers, including significant travel times to care, absence of reliable transportation, and lack of reliable internet access for telehealth, further hinder access. For individuals who can surmount some or even all these barriers, clinician and physician shortages still impede access to care, with the majority occurring in rural settings [7].

The state of Oregon – in the Pacific Northwest region of the US – and its rural communities are no exception to the rural health penalty. People living in rural Oregon have a higher mortality rate and higher levels of chronic diseases than people living in urban Oregon [8, 9]. Factors that perpetuate this rural–urban health disparity include important social determinants of health, such as larger portion of rural residents living in poverty, receiving less education, and experiencing higher unemployment rate [9]. Health system inequities, such as lower rates of chroic disease screenings, peretuate health disparities leading to high numbers of emergent and non-emergent utilization of emergency departments (ED) across the state [10, 11].

Mobile health clinics (MHCs) have been implemented as an intervention to address growing health inequities and the number of barriers to accessing health care [12]. With over 1,000 MHCs implemented across the US, the most common services provided are child immunizations, flu shots, blood pressure screenings, obesity screenings and vision screenings [13].

MHCs have been found to (1) reduce patient travel needs and prices for care and (2) improve health outcomes among the communities by increasing screenings, enabling early diagnosis of chronic conditions, and providing health counseling for disease management and prevention [12]. Through accessible screenings, evidence suggests that MHCs identify and treat high rates of previously undiagnosed hypertension, diabetes, cancers, and hypercholesterolemia in study populations [14–16]. With accessible follow-up care and counseling services integrated into the MHCs, there is a high adherence rate to follow-up counseling and subsequent reduction in patient hypertension [15]. In addition, MHCs can deliver essential services to key populations in the US that are vulnerable to the barriers in accessing quality care and experience various health disparities, with the majority of patients being women and racial/ethnic minorities in both primary care and preventative care outreach [16]. Thus, MHCs as alternative points for seeking medical care have the potential to ameliorate the current rural–urban health disparity in chronic diseases, access key rural populations, and have downstream impacts on the health system by reducing the number and cost of emergent and nonemergent ED visits.

Previous cost analysis on MHCs have calculated return on investment (ROI) of MHCs, cost-effectiveness of screening tools, and cost analyses of operating and maintenance costs [17–19]. A survey-based cost analysis of 96 MHCs in the US found that the average cost per patient visits in a variety of MHC services ranges from 65 to 529 USD with mean operating costs of 300,000 USD to 2.5 million USD [19]. The main estimated costs of MHCs reside with startup costs and maintenance of the mobile unit, particularly in the face of poor weather conditions and vehicle breakdowns [12]. Studies estimated 2.5 million USD cost reduction in avoided ED visits annually in Southern California and an overall ROI of $23 for every $1 invested in a mobile clinic [12, 15, 16]. In France, evidence suggest that mobile mammography units is cost-effective with an incremental cost per additional screen of €610 (95% CI, 492 to 821), and the incremental cost effectiveness ratio (ICER) becoming even more favorable in more remote areas [18].

While these studies present the beginnings of cost analysis of mobile interventions, they are restricted in their application to the US rural–urban health disparities. For instance, they fail to distinguish outcomes specific to primary care in rural settings and outcomes between patient sub-populations; furthermore, these studies do not provide insight into the time motion of providers. In addition, there is a lack of analysis on human resource allocation required of a MHC provider and of a CHW, which may be essential in various low-source settings that serve vulnerable populations – as human resources have been found to be one of the largest cost factors [19].

It is critical to understand the detailed cost of MHCs and the full resource allocation involved to ensure feasibility of operation to inform evidence-based decision-making – as health systems increasingly view MHCs as an effective intervention. Namely, in the US, the Maximizing Outcomes through Better Investments in Lifesaving Equipment for (MOBILE) Health Care Act, passed in 2022, allowing federal funds allocated to community health centers in rural and underserved areas to be used to establish mobile clinics [20]. Increasing understanding of the cost and resource allocation necessary to operate MHCs in the US will enable consistent resource allocation to these resource limited settings with knowledge transfer facilitating successful implementation.

The objective of this study is to generate evidence on the cost of full operations and utilization of a rural MHC that reaches a diverse patient population with equally diverse medical needs, highlighting the integral work of CHWs in the MHC operations to meet patient medical and social needs. To achieve our objective, we conducted a cost analysis to evaluate the cost of a rural mobile clinic and a time motion study (TMS) to assess human resource allocation across different rural communities. The findings may inform health systems interested in implementing a MHC intervention to serve rural communities.

## Methods

### Intervention

In Oregon, La Clínica is a MHC intervention that provides care to rural communities including people experiencing houselessness (PEH), Native Americans, and migrant and seasonal farmworkers, along the Hood River. This MHC model was initiated to address rural health disparities and barriers to accessing health care services, as access to urgent, emergent, and primary care services for patients in this setting is geographically restricted. The MHC does so by providing chronic health screenings, urgent care, and mental health care at a sliding-scale discount [10]. Staff on the MHC include a nurse practitioner (NP) and a medical assistant to provide medical care, and a community health worker (CHW) to complete outreach with the community and assist with patients’ non-medical needs.

### Study design

This cost analysis used retrospective data from existing business ledgers, electronic medical records, and previously reported literature to evaluate the cost of a rural mobile clinic [21]. A TMS was conducted to assess human resource allocation of CHW and provider time within a rural MHC setting and to adjust for potential discrepancies in the use of current procedural terminology (CPT) codes between MHC visits.

The cost analysis was conducted from the health system perspective. Health system provider perspective considered direct medical costs and direct non-medical costs to understand the resources needed for the health system to provide and sustain the healthcare intervention. The time horizon, referring to the duration that costs and procedure utilization were measured, was one year. This year included patient care seeking behavior during the intervention period, between April 2022 to April 2023. Due to a horizon of one year, there was no discounting of costs or benefits. Cost data was assessed for the 2022–2023 period.

### Setting

The analysis sample included patients who were residing within either Wasco County or Hood River County, sought medical care at La Clínica Mobile Unit between April 2022 to April 2023, and have available electronic medical records. Wasco country is a rural, non-metropolitan area with a population of 26,736 in 2021 [22]. Most recent estimates in January 2022 reported nearly 200 people experiencing houselessness in this county [23]. Wasco county has 13.3% of its population in poverty, which is 1.1% higher compared to Oregon state averages and 1.7% higher compared to national averages [22]. Additionally, Wasco County reported 11.4% of persons living without insurance compared to 7.3% in Oregon state and 9.8% in the US [22].

Hood River county is a rural, non-metropolitan area with a population of 24,057 in 2021 [24]. Most recent estimates in January 2022 reported nearly 71 people experiencing houselessness in this county, a reported 27% increase since 2020 [25]. Hood River county has 10.9% of its population in poverty, which is lower compared to Oregon state averages and national averages [24]. Additionally, Hood River County reported 14.4% of persons living without insurance compared to 7.3% in Oregon state and 9.8% in the US [24].

Access to urgent, emergent, and primary care services for patients in both counties was previously described as geographically restricted. There is one hospital located in the northern county with emergency services, primary care services, as well as an urgent care with limited operating hours. To meet the needs of the population, La Clínica mobile clinic was established in April 2022 by the not-for-profit organization One Community Health [26].

### Analytic approach

We conducted a cost analysis and included a TMS to understand providers’ time allocation at the MHC. The MHC served different population-based sites based on locations with high density of different communities, including Native American in Lieu sites, a temporary shelter and soup kitchen for those experiencing houselessness, two isolated rural communities, and migrant/seasonal farmworker sites near packing houses and local orchards. These sites resulted in four distinct rural sub-populations targeted by the MHC. Descriptive statistics were used to analyze and describe patient demographics for the MHC rural patient populations via R statistical software, which was categorized into four sub-populations based on outreach efforts by the MHC at rural locations with a high-density of: Isolated rural dwelling, PEH, migrant or seasonal farmworker, or Native American [27]. Isolated rural dwelling population was used as a baseline comparator, defined as a patient who received care at a site that was not high-density for people experiencing houselessness, migrant or seasonal farmworkers, and Native Americans. Sociodemographic information about the patient population was categorized and analyzed to illustrate statistical differences in each sub-population, using both chi-square analysis and Fisher’s exact test. Detailed reporting of the analysis is included in Additional File 1.

#### Cost and resource outcome

Measurements and valuation of resources and costs were assessed in USD for the year 2023 from the perspective of the provider. Costs considered were direct operating costs of the mobile clinic including clinic operations, resources, staffing, and staff time resource over one calendar year. Additional costs collected included startup cost for the rural MHC, including the cost of the unit, necessary refurbishments, and durable medical and nonmedical equipment. The costs were assigned as either a start-up cost or an operating cost. Quantities and cost of both startup costs and operating costs were obtained from clinic business ledgers for the calendar year of 2022. Startup costs included purchase and refurbishment of the MHC Unit, durable medical supplies, legal costs, and other one-time necessary purchases as recorded in the clinic business ledger. Operating costs were included for the calendar year 2022 and included staff salaries, maintenance costs, outreach costs, repeated medical supplies, and operating costs, such as gas, propane, and WIFI.

#### Healthcare utilization

Patient resource utilization was assessed using CPT procedure codes from MHC patient records. The procedure codes were assigned a cost value based on the Oregon Health Plan 2023 fee-schedule [28]. The procedure codes were analyzed for frequency, overall and by sub-population. Chi-square analysis was used to compare CPT utilization by patient sub-populations. Detailed methods are included in Additional File 2.

### Time motion study

A TMS was conducted to collect activity-based data for calculation of human resource cost for MHC clinical provider, a NP, and MHC CHW. The data was acquired for a minimum of three shifts observing human resource allocation at two sites with a high-density of patients. These two sites included those who are isolated rural dwelling and those experiencing houselessness.

#### Pilot testing

Piloting of the TMS was performed over four days in which direct observation of the participants resulted in refining the data collection tool and the developed task categories. A tablet tool was used to record all entries using the mobile application Toggl. Task categories were based on previous time and motion studies conducted by Overhang et. Al. (2001) and adapted to meet the needs for the MHC, including addition of MHC related tasks, addition of multitasking tasks, and removal procedural tasks not conducted on the MHC. The analysis groupings piloted by Overhang et. Al. (2001) were used to associated tasks between direct patient care, indirect patient care, administration tasks, miscellaneous tasks, and MHC specific tasks.

During the study period, the impact and utilization of the MHC services on the community was observed to expand beyond just the medical care provided by the NP and medical assistant. Participation and task categories were expanded to include the role of CHWs in MHC interventions. Specifically, investigating the distinct role of the CHW in addressing non-medical needs, coordinating patient care, and expanding MHC involvement in the community. For instance, it was observed that some patients would come in to use these CHW services alone and receive no medical care from the medical staff.

#### Participants and observation procedure

Study participants were the providers working on the MHC: an NP and CHW. Informed consent was obtained prior to observation. Work shadowing observations were conducted from May 2023 through June 2023 for twelve shifts over six weeks accounting for 40 h of direct observation of each participant. The observer was a study researcher (co-author AH) from an external organization piloted, CITI certified, and trained using the application Toggl for data collection. The observer followed each participant for the duration of their shift. Observations began at 8:30am each morning with a focus on the activities performed during the time spent on the mobile unit. Observations ended each day with the termination of activities on the MHC upon returning to the base clinic. After each observation period, participants were asked to self-report any work activities that resulted outside of the period of observation. Patients and patient families present were informed of the study and that consent for observation during each visit was voluntary. Consent was obtained verbally to protect patient privacy.

#### Data collection

Direct patient care activities were defined as any time spent in direct communication with the patients. Indirect patient care activities were defined as engaging in activities that impacted patient care but was not in direct communication with the patient, such as time spent charting, writing notes, placing orders, talking with colleagues, searching for individuals, and searching for supplies for patients. MHC related activities were defined as the time spent on MHC specific needs including travel time, setting up, and talking to colleagues about MHC specific needs. Full list of tasks and their coded category is included in Additional File 3.

Additional categories were assigned and analyzed for the CHW role due to their distinct roles from a medical provider. These included MHC specific tasks, patient medical care tasks, patient social determinants of health (SDOH) tasks, and finally, patient care coordination via interprofessional collaboration. MHC specific activities were directly attributed to operating or managing the MHC such as driving the unit, ordering parts/maintenance on the vehicle, and managing the staffing/admin meetings with the MHC. Medical care tasks included checking patients in, looking for medical supplies, charting patient encounters, and talking/educating the patient about health information. Patient SDOH tasks included assisting patients with any social need, such as housing insecurity, insurance, and food insecurity. Care coordination included tasks that requiring collaboration with transdisciplinary services or providers and included collaboration with community partners, other clinics, and individual patient care needs. Further breakdown of these categories is included in Additional File 3.

#### Data analysis

Tasks were categorized into analytic categories and summarized by percent time, mean time in minutes, and standard deviation. This was done for each participant’s observed time overall and among each patient sub-population. Percent time spent on each category was compared for NP and CHW time between isolated rural dwelling patients and PEH sub-populations using Fisher’s exact test, due to small cell counts. The most frequent tasks completed within each analytic category were documented and compared between sub-populations using Fisher’s exact test [29]. Additional description of CHW specific analytical categories were presented as percent time per shift. Finally, NP time per patient visit was estimated and presented with the most frequent tasks completed, analyzed as percent time by specific task. A t-test analysis compared isolated rural patients and PEH time per patient visit with NP.

## Results

### Cost inputs

Startup costs were collected from retrospective business ledgers to assess necessary refurbishments and durable medical equipment purchases. The economic value of the RV was collected from expert stakeholder opinion and based on market values from Kelley Blue Book in 2023. In total, startup costs accumulated to be 275,625 USD. The highest startup cost for the MHC was dedicated to the RV unit itself, accounting for 92% of startup costs. The summary of annual operating costs and startup costs for the rural MHC are presented in USD 2023 in Table 1. Operating costs for one calendar year (CY) – derived from the business ledger – were estimated to be 308,470 USD. Three full time staff salaries accounted for 75% of annual operating costs and vehicle maintenance accounted for 23% of annual operating costs.

**Table 1:** Start up and operational costs of rural mobile health clinic, 2023

Major Category	Description	Cost USD 2023	Source	Start-up or Operational
MHC Unit	RV (New), RV (current value), and Vehicle Refurbishments	252 922.50	Stakeholder Expert Opinion, Kelley Blue Book Market Value, & Ledger CY 22	Start Up
Parked Premises	Cost of renting use of public land for 4 days a week for 48 weeks	Donated 0.00	Stakeholder Expert Opinion	Operational
Staff	Three full-time employees	232 200.70	Ledger CY 22	Operational
Outreach	Includes the price of flyers, printing labels, and MHC labeling	2 593.41	Ledger CY 22	Operational
Medical supplies	Includes waste disposal, procedural supplies, durable small equipment, testing supplies and professional license	1 461.21 (Operational) 12 469.54 (Start Up)	Ledger CY 22	Operational & Start Up
Procedures	Cost of procedures (Average cost per patient encounter)	27 518.02 (Avg. 13.89)	CPT Fee Schedule	Procedural
Other Operating costs	Gas, propane, Wi-Fi, janitorial contract, vehicle repair and maintenance	72 214.69	Ledger CY 22	Operational
Other Costs	Includes cost of legal fees, grant applications, and trainings	10 233.72	Ledger CY 22	Start Up
**Total Economic Value**:	584 095.76 USD	**Start Up Value**:		275 625.76 USD
		**Operational Value:**		308 470.00 USD

Economic cost of startup and one calendar year of operation for rural MHC in USD 2023. Broken down by category and assigned as an operational or startup cost. Description of each category and source of information is noted in individual columns

*CY22* Calendar Year 2022, *MHC* Mobile health clinic

### Time Motion Study (TMS)

#### Nurse Practitioner (NP)

We collected 48 h of observational data on MHC NP time use over six shifts. Three shifts were accessible for PEH and three were accessible for isolated rural communities. During the observation period 42 patients were seen by the NP. Overall, 32% of NP time was spent in direct patient per shift (mean = 153.00 min (SD = 37.80 min) and 38% of NP time was spent on indirect patient care per shift (186.60 (53.40)). Additionally, 21% of NP time overall was spent on MHC specific activities (104.22 (SD 23.90)). See Table 2. NP patient care time did not vary significantly between the two sub-populations by analytical category (Fisher’s, *p* > 0.01), however the type of tasks within each analytical category did vary between sub-populations for every category (Fisher’s, *p* < 0.01). See Table 3.

**Table 2:** Average provider time (in Minutes) spent per shift on patient care, overall and by sub-population

**Nurse Practitioner**			
**Task Category**	**Overall *N* = 6** **Avg. Min. (SD)**	**Isolated Rural *N* = 3** **Avg. Min. (SD)**	**PEH *N* = 3** **Avg. Min. (SD)**
Direct Patient Care	153.00 (37.80)	126.42 (29.46)	180.18 (24.30)
Indirect Patient Care	186.60 (53.40)	222.18 (45.36)	150.60 (35.34)
Administration	29.52 (33.78)	21.96 (31.01)	37.14 (41.58)
Miscellaneous	12.78 (19.08)	26.36 (20.64)	3.18 (2.04)
Mobile Health Clinic	104.22 (23.94)	103.62 (33.00)	104.80 (18.16)
**Community Health Worker**			
**Task Category**	**Overall *N* = 5** **Min (SD)**	**Isolated Rural *N* = 2** **Min (SD)**	**PEH *N* = 3** **Min (SD)**
Direct Patient Care	61.13 (40.90)	31.17 (32.7)	81.10 (36.26)
Indirect Patient Care	136.68 (73.52)	67.92 (31.73)	182.51 (49.28)
Administration	32.40 (59.41)	72.27 (93.89)	5.81 (1.06)
Miscellaneous	27.14 (26.61)	42.66 (41.58)	16.80 (12.28)
Mobile Health Clinic	182.02 (29.46)	209.34 (3.65)	163.81 (22.03)

Mean number of minutes rural mobile clinic provider spent on direct patient care tasks, on indirect patient care tasks, on administration tasks, on miscellaneous tasks, and on mobile health clinic administration tasks/travel time. Mean time was calculated overall, and by each distinct rural sub-population

*N* Number of shifts observed, *PEH* Patient experiencing houselessness

**Table 3:** Percentage of rural mobile clinic provider time spent on type of tasks overall and by patient sub-population

**Nurse Practitioner**				
**Analytic Category**	**Overall %**	**Isolated Rural %**	**PEH %**	***p*-value > 0.01**
Direct Patient Care	32%	13%	19%	< 0.01
Indirect Patient Care	38%	23%	15%	< 0.01
Administrative	6%	2%	4%	< 0.01
Miscellaneous	3%	2%	0%	< 0.01
Mobile Health Clinic	21%	11%	11%	< 0.01
**Community Health Worker**				
**Analytic Category**	**Overall %**	**Isolated Rural %**	**PEH %**	***p*-value < 0.01**
Direct Patient Care	14%	7%	18%	< 0.01
Indirect Patient Care	31%	16%	41%	< 0.01
Administrative	7%	17%	1%	< 0.01
Miscellaneous	6%	10%	4%	< 0.01
Mobile Health Clinic	41%	49%	36%	< 0.01

Percentage of time each Rural mobile health clinic provider spent on each type of tasks during time motion study. Separated to include time overall, and specifically for isolated rural patient populations and rural PEH. Fisher’s Exact test compared the analytic category between each sub-population. Additionally, the percentage of time spent on specific tasks within each category was compared using Fisher’s Exact test between isolated rural patients and rural PEH. Significance was set at 0.05

*PEH* Patients experiencing houselessness

**Fig. 1:**
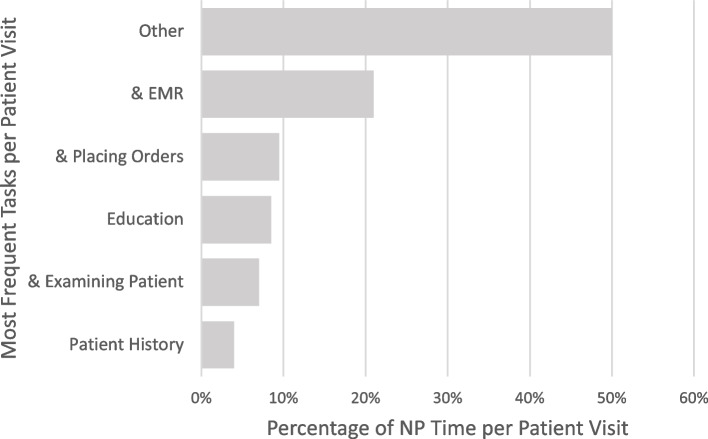
Proportion of Time (in Minutes) on Task per Patient Visit with NP, Overall. Average proportion of nurse practitioner time (in minutes) spent on activity per each patient visits, broken down to show the five most common activities. “&” Indicates that the provider was multitasking while talking to patient. For example, “& EMR” indicates that the provider was talking to patient and reading the electronic medical record (EMR). EMR = Electronic Medical Record; NP = nurse practitioner

Average NP time spent per patient visit (Fig. 1) was 17.40 min (mean = 17.40 min (SD = 1.38 min)) per PEH visit, compared to 34.52 min (34.52 (10.14) per isolated rural patient visit (t-test, *p* > 0.01). Talking to the patient and working on the electronic medical record (EMR), placing orders, patient education, physical exam, and obtaining a patient history were the most frequent tasks performed during patient visits. See Fig. 1.

### Community Health Worker (CHW)

We collected 36 h of observational data on MHC CHW time use over five shifts. Three shifts were accessible for PEH and two were accessible for isolated rural communities. CHW time spent per shift was reported between analytic categories in minutes (Table 2). Overall, 14% of CHW time was spent on direct patient care (mean = 61.13 min, SD = 40.90 min) and 31% of time on indirect patient care (136.68 (73.52)). The CHW spent 41% of time on MHC specific activities (182.02 (29.46)). See Table 3. Percent of CHW time spent by analytical category with patient sub-populations varied significantly (Fisher’s, *p* < 0.01). Additionally, the type of tasks within each analytical category varied between sub-populations for every category (Fisher’s, *p* < 0.01). See Table 3.

#### CHW specific analytic categories

**Fig. 2:**
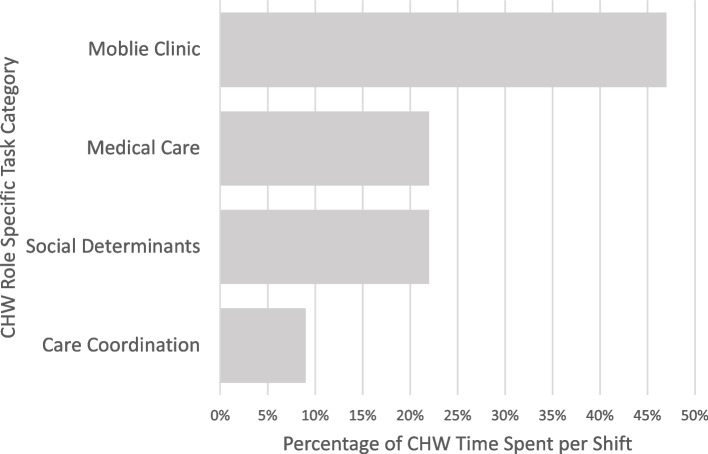
Proportion of Community Health Worker Time (in Minutes) on Patient Care Task, Overall. Percent community health worker (CHW) time spent on role-based tasks per shift. Roles are separated into four general labels. Mobile Clinic (MHC) (47%) = Mobile health clinic tasks: Time spent on activities directly attributed to managing or operating the MHC. Medical Care (MC) (22%) = Medical care: Time directly assisting with patient medical care. Social Determinants (SDOH) (22%) = Social Determinants of Health: Time assisting patients with any social need that is not medical care. Care Coordination (PCC) (9%) = Patient care coordination: Time collaborating with transdisciplinary services or providers

A significant amount CHW time (47%) was spent on MHC specific tasks (mean = 182.02 min (SD = 29.46 min)). CHW time spent on patient medical care tasks allocated 22% of time per shift (85.05 (81.97)). Another 22% of CHW time was spent on patient social determinants of health (SDOH) tasks (87.66 (86.71)). Finally, 9% of CHW time was spent on patient care coordination via interprofessional collaboration (35.79 (25.77)). Percentage of CHW time on medical care and SDOH tasks were found to vary significantly when comparing isolated rural patients and PEH, with more time spent on these activities with PEH (Fisher’s, *p* < 0.01). Activities not included in these roles include miscellaneous tasks and other administrative tasks (Fig. 2).

### Procedure utilization

A total of 232 distinct days of patient visits on the MHC were included in the analysis. Over the time period, 1,981 patient encounters with 812 unique patients were recorded and included in the analysis. Significant differences were found between sub-population demographics and use of MHC procedures. The detailed breakdown of patient demographics and procedure utilization is provided in Additional File 1 and 2.

#### Sociodemographic information

Patients from Isolated rural communities were found to have the largest proportion of older adults (13%) and the largest proportion of patients on private health insurance (25%). PEH were found to have the largest portion of White, male patients, aged 18–65, with 69% of the sub-population on Medicaid insurance. The migrant or seasonal worker population had 92% of patients speaking Spanish and the largest proportion of patients on self-pay insurance (56%), with 20% having no insurance. Finally, most Native American patient population was found to be on Medicaid insurance (69%), male (56%) and English speaking (99%).

#### Frequent procedures utilized

Of the procedures utilized on the MHC, 31% of reported procedures were vaccinations (*n* = 438, 31%), 27% were Covid-19 related encounters (*n* = 377, 27%), 13% were outside referrals (*n* = 179, 13%), and 12% were point of care testing (POCT). Significant differences in utilization of these frequent procedures were found between sub-populations (χ^2^, *p* < 0.01) with Isolated Rural patient use dominated by Covid-19 and Influenza vaccines whereas PEH use was dominated by point of care STI testing. Detailed breakdown of each patient population procedure utilization is included in Additional File 2.

## Discussion

With the recent passing of MOBILE Care Act in 2022, understanding the complex cost and utilization of MHCs is essential for successful implementation and improved community health, particularly in a rural setting [20]. The case study provides insight into the operations of a preventative and primary care MHC in a rural setting that is transitioning out of the Covid-19 pandemic. Specifically, the findings contribute to understanding providers’ time use across different vulnerable populations within the context of overall cost to generate evidence that may aid future implementation strategies.

This study consisted of a retrospective cost analysis, including a TMS of a preventative/primary MHC in a rural setting. The study found that overall costs align with nationally reported ranges and human resource allocation, which has yet to be investigated in this setting, differed significantly between target populations for the CHW. The findings suggest that when targeting key populations and engaging hard-to-access patients with a MHC intervention, health care resources and human resource allocation will vary across sub-populations.

Understanding how resources are allocated within a rural mobile clinic intervention from a health system perspective is critical as rural access to care is important for national and local policymakers. Associated start-up and operational costs for the preventative care mobile clinic intervention are reflective of previous published literature, dominated by the cost of the clinic itself and cost of staffing, respectively [12, 19]. Large startup costs continued to present a barrier to implementation and continued operation in underserved communities due to a lack of consistent funding [12].

To understand the way that rural healthcare resources were used within the MHC intervention, we investigated the retrospective procedure utilization and human resource allocation. Procedures such as influenza vaccine, blood glucose point of care testing, Covid-19 vaccines, and STI testing dominated the utilization by all rural patients, indicating a combination of usage for both management of chronic diseases and urgent care needs. Additional, non-medical resources were also addressed by the MHC CHW staff, including insurance needs, housing needs, transportation needs, and continuity of care/referral to higher level services.

Looking beyond annual funding and procedure utilization of the intervention, we found that human resource allocation varied between key populations. Specifically, PEH and isolated rural patients differed in their utilization of NP time, CHW time, point of care testing, and covid-19 related procedures. When working with PEH population, the NP spent a larger portion of their shift on direct patient facetime, and less time per patient visit, however this did not vary significantly. This pattern is likely due to the larger number of PEH seen per shift, compared to other isolated rural populations, highlighting the impact of population density on use of MHC services. On the other hand, when working with PEH, the CHW spent significantly more time per shift on direct and indirect patient care, completing medical care tasks and addressing the social needs of patients. This difference is partially attributed to the higher average number of patients seen per shift in days completing outreach to PEH. However, this is also attributed to the higher burden of social needs experienced by PEH, who experience a cross section of health disparities and social inequities due to place and housing insecurity. This intersection of need, particularly addressing SDOH, was able to be addressed by the CHW, who’s breadth and depth of contributions included organizing housing assistance, ride assistance, insurance assistance, and more for MHC patients [31].

This study contributes to the current acknowledgement of the work completed, and contribution to patient care made, by CHWs. CHWs who are familiar with the community may be essential in reaching hard to reach population. However, their work is potentially undervalued by the current health system. In this TMS, the CHW working on the MHC was able to access various populations and expand community health services by addressing patient SDOH, collaborating with patient medical care, and interprofessional collaboration between the MHC intervention and community partners. Research has shown the ability of CHW programs to reduce chronic diseases and lower health system costs by reducing ED utilization and hospital stays – an impact mirrored by MHC interventions [32–34]. Despite these documented contributions by CHWs to patient care in previous literature and in this study, there was a lack of billing codes to assess CHW impact retrospectively. Additionally, as little as half of US state Medicaid programs provide any form of financing for CHW services [32]. This is significant because human resource costs presented as one of the largest cost barriers for continued operation of MHCs and brings into consideration a need for improvements in renumeration for CHW time.

The MHC intervention is a prime example of the collaboration necessary between medical care, social needs, and cultural competency required to assist members of the community with elevated risk for worsening chronic disease – an intersection which CHWs can navigate with expertise. Needs for consistent integration and funding of these services spark a conversation about policy needs, continued research, and proper allocation to CHW work.

Chronic diseases are degenerative, they are persistent and long-lasting, and they require consistent management over years to prevent deterioration of one’s health. For rural communities, the burden of these chronic diseases has already been found to be larger than on the urban communities, and despite a lower population density, rural communities still saw a huge diversion of resources, medical encounters, and increased negative social implications [35]. The services provided by the MHC indicate levels of increased access to populations that may not have sought care or accessed care easily prior to the intervention.

### Limitations

This study evaluated the cost and resource allocation required to operate a rural preventative and primary care MHC that accesses key populations, with several limitations acknowledged. First, the study is a case study of a population accessing care at a single mobile unit, which creates limited generalizability. Findings were compared to national surveys of MHCs and understood to be an addition to the current literature. Due to the size of the MHC, we have a small sample size for the TMS study (one provider, one CHW). However, this study is the first to collect data on provider time use and to understand the role of different health care professionals in a mobile clinic setting, opening the door for future work. Lastly, the generation of sub-populations within this case study was done based on pre-assigned outreach days by the MHC to each population. This limits the ability to concretely acknowledge the intersectionality of patient needs in a rural setting. However, this also highlights the importance of reaching the health needs of such key populations in rural settings that sit at the intersection of place and social health inequities [30].

## Conclusion

The MHC is a healthcare model that can impact rural community health and the health system through improved health outcomes of multiple populations. In the rural setting where this study took place, the MHC provides a conduit to accessing populations, managing chronic diseases, and engaging patients in care. When comparing two of these patient cohorts (isolated rural patients and PEH) there are differences in their insurance rates, human resource allocation, and utilization of MHC services. These findings present a descriptive understanding of how MHCs operate differently with different patient populations. Additionally, this study provides insight into the impact of a CHW within a community-based intervention. As local policy makers consider the resources available and the health needs for their community, a complete understanding of the resources required to operate an MHC is essential for informed decision making and successful implementation in underserved communities that would benefit from increased access to healthcare.

## Supplementary Information

Supplementary Material 1. Sociodemographic Information. Description: Contains the methods and results breakdown of the patient population that utilized mobile health clinic services over the study time period. Includes breakdown and descriptive statistical analysis of the patient’s insurance status, emergency department use, and demographics, including comparisons between the four patient sub-populations (Rural patients, patients experiencing houselessness, migrant or seasonal workers, and Native American patients). Supplementary table 1 and Supplementary table 2 presents the information in table format.

Supplementary Material 2. Healthcare Utilization of Patient Sub-Populations. Description: Contains the methods and results breakdown of the patient procedure utilization of mobile health clinic services over the study time period. Includes breakdown and descriptive statistical analysis of the most frequent services provided by the mobile clinic and comparisons between the two patient sub-populations (Isolated Rural patients and patients experiencing houselessness). Supplementary table 3 presents the most common procedures utilized and their Medicaid cost information in table format. Supplementary figure 1 presents the change in use of the mobile clinic across two time periods, the first being the first year of operation, aligning with peak pandemic use and the second period being the second year of operation, noted as less dominated by the covid-19 pandemic, and colloquially referred to as “post” pandemic, with the acknowledgement that the definition is a transition term – not an absolute. Supplementary figure 2 and 3 compare the type of procedures used during covid and “post” covid by rural patients and patients experiencing houselessness, respectively.

Supplementary Material 3. Full List of Time Motion Activities. Description: Includes list of activities for the task and analytic categories used during the time motion data collection for both the nurse practitioners and community health workers as well as their defined category for analysis.

Supplementary Material 4. Supplementary Figure 1: Top procedure codes utilized on Rural mobile health clinic, 2022 to 2023.

Supplementary Material 5. Supplementary Figure 2: Change in Rural patients procedure utilization between two time periods.

Supplementary Material 6. Supplementary Figure 3 Change in patients experiencing houselessness procedure utilization between two time periods. 

## Data Availability

The datasets used and/or analyzed during the current study are available from the corresponding author on reasonable request.

## Abbreviations

US: United States
ED: Emergency Department
MHC: Mobile Health Clinic
ROI: Return on Investment
ICER: Incremental Cost Effectiveness Ratio
MOBILE Act: Maximizing Outcomes through Better Investments in Lifesaving Equipment for (MOBILE) Health Care Act
FQHC: Federally Qualified Health Center
CPT: Current Procedural Terminology
PEH: Patient Experiencing Houselessness
NP: Nurse Practitioner
CHW: Community Health Worker
CY: Calendar Year
POCT: Point of Care Testing
SDOH: Social Determinant of Health
TMS: Time Motion Study
