# Supplementary material for: Mobile health clinics in a rural setting: a cost analysis and time motion study of La Clínica in Oregon, United States
Source: BMC Health Serv Res. 2025 Jan 17;25:97. doi: 10.1186/s12913-024-12203-5 (PMC11740325; doi:10.1186/s12913-024-12203-5)
Supplement: Supplementary file 1 — Supplementary Material 1. Sociodemographic Information. Description: Contains the methods and results breakdown of the patient population that utilized mobile health clinic services over the study time period. Includes breakdown and descriptive statistical analysis of the patient’s insurance status, emergency department use, and demographics, including comparisons between the four patient sub-populations (Rural patients, patients experiencing houselessness, migrant or seasonal workers, and Native American patients). Supplementary table 1 and Supplementary table 2 presents the information in table format. [file 12913_2024_12203_MOESM1_ESM.docx]

**Additional File 1: Sociodemographic Information**

**Methods**

The MHC served different population-based sites, including Native American in Lieu sites, temporary shelter, and soup kitchen for those experiencing houselessness, two isolated rural communities, and migrant/seasonal farmworker sites near packing houses and local orchards. These sites resulted in four distinct rural sub-populations accessed by the MHC: isolated rural dwelling, patients experiencing houselessness (PEH), Native American, and migrant or seasonal worker. Patient race and ethnicity, age, insurance status, language spoken, sex, and emergency department (ED) use varied significantly by population.

The following variables were included in the analysis: race/ethnicity, age, insurance, patient language spoken, sex, and emergency department use via R statistical software. Each sub-population was analyzed separately and presented as counts with percentages. The isolated rural sub-population was then compared with the PEH sub-population, due to their inclusion in the time motion analysis. Analysis was done with both chi-square and Fisher’s exact test to ensure validity of the test due to low cell counts but tables larger than 2x2 tables. Sex was separated into male or female based on limited labels used in the medical chart. Age was separated into four groups, 0-17, 18-44, 45-64, and 65+. Race and ethnicity were separated into four groups (White, Hispanic/Latinx, Asian, and American Indian or Alaskan Native). Insurance status was separated into five groups (Medicare, Medicaid, Private Insurance, Self-pay, Uninsured). Uninsured was defined as those with no insurance, however, were covered under a special program or grant. Whereas Self-pay was defined as no insurance and no coverage under a special program or grant. The number of patient ED visits from May 2021 to May 2023 was collected and aggregated using the electronic medical record system. ED use was separated into four groups (no use, some use, frequent use, and high frequency use) and compared.

**Results**

Isolated rural dwelling patients were found to have the largest proportion of older adults (13%) and the largest proportion of patients on private health insurance (25%). PEH were found to have the largest portion of White, male patients, aged 18-65, with 69% of the sub-population on Medicaid insurance. The migrant or seasonal worker population had 92% of patients speaking Spanish and the largest proportion of patients on self-pay insurance (56%), with 20% having no insurance. Finally, most Native American patient population was found to be on Medicaid insurance (69%), male (56%) and English speaking (99%). Overall, most patients had no ED use, with a few outliers extending to more than seven visits to the ED. The patient demographic breakdown is reported in Supplementary Table 1.

Comparison of demographics between Isolated Rural patients and PEH were all found to be significant for chi-square and Fisher’s exact tests (p < 0.01), except for ED use. Supplementary Table 2.

| **Supplementary Table 1:** Patient Demographic Breakdown of Rural Mobile Health Clinic | | | | | |  |
| --- | --- | --- | --- | --- | --- | --- |
|  | **Total Seen at MHC** | **Isolated** | **PEH** | **Migrant or Seasonal** | **Native American** |  |
| ***Variables*** | *N (%)* | *N (%)* | *N (%)* | *N (%)* | *N (%)* |  |
|  | *N* **= 812** | N=544 | *N=81* | *N=161* | *N=71* |  |
| **Race and Ethnicity** |  |  |  |  |  |  |
| White | 277 (41%) | 204 (46%) | 57 (86%) | 7 (5%) |  |  |
| Hispanic or Latinx | 323 (48%) | 178 (40%) | 4 (6%) | 129 (92%) |  |  |
| Asian | 4 (0.6%) | 4 (1%) | 0 (0%) | 0 (0%) |  |  |
| Native American | 71 (11%) | 62 (14%) | 5 (8%) | 4 (3%) |  |  |
| **Age** |  |  |  |  |  |  |
| 0-17 | 133 (16%) | 116 (21%) | 0 (0%) | 9 (6%) | 14 (20%) |  |
| 18-44 | 352 (43%) | 187 (34%) | 41 (49%) | 111 (69%) | 25 (35%) |  |
| 45-65 | 242 (30%) | 170 (31%) | 38 (45%) | 33 (21%) | 24 (34%) |  |
| 65+ | 85 (11%) | 71 (13%) | 5 (6%) | 8 (5%) | 8 (11%) |  |
| **Insurance** |  |  |  |  |  |  |
| Medicaid | 321 (40%) | 242 (44%) | 58 (69%) | 21 (13%) | 49 (69%) |  |
| Medicare | 65 (8%) | 51 (9%) | 12 (14%) | 2 (1%) | 4 (6%) |  |
| Private | 152 (19%) | 137 (25%) | 1 (1%) | 14 (9%) | 7 (10%) |  |
| Self-pay | 223 (27%) | 98 (18%) | 12 (14%) | 90 (56%) | 9 (13%) |  |
| Uninsured | 50 (6%) | 16 (3%) | 1 (1%) | 33 (20%) | 2 (3%) |  |
| **Language Spoken** |  |  |  |  |  |  |
| English | 491 (60%) | 387 (71%) | 83 (99%) | 12 (7%) | 70 (99%) |  |
| Spanish | 312 (38%) | 151 (28%) | 1 (1%) | 148 (92%) | 0 (0%) |  |
| **Sex** |  |  |  |  |  |  |
| Male | 478 (59%) | 271 (50%) | 57 (68%) | 132 (82%) | 31 (56%) |  |
| Female | 334 (41%) | 273 (50%) | 27 (32%) | 29 (18%) | 40 (44%) |  |
| **ED Use** |  |  |  |  |  |  |
| No use (0 visits) | 730 (90%) | 478 (87%) | 72 (89%) | 154 (96%) | 65 (92%) |  |
| Some use (0-3 visits) | 63 (8%) | 52 (10%) | 4 (5%) | 7 (4%) | 5 (7%) |  |
| Frequent use (4-6 visits) | 10 (1%) | 7 (1%) | 3 (4%) | 0 (0%) | 0 (0%) |  |
| High Frequency use (7+ visits) | 9 (1%) | 7 (1%) | 2 (2%) | 0 (0%) | 1 (1%) |  |
|  |  |  |  |  |  |  |
| Descriptive presentation of rural MHC patient demographics, separated by patient sub-population. | | | | | |  |
| MHC = Mobile Health Clinic; PEH = Patients Experiencing Houselessness | | | | | |  |

| **Supplementary Table 2: Comparison of Demographics Between Rural Mobile Health Clinic Isolated Rural Patients and Patients Experiencing Houselessness** | | |
| --- | --- | --- |
|  | **Isolated** | **PEH** |
| ***Variables*** | *Chi-Square* | *Fisher* |
|  |  |  |
| **Race and Ethnicity** | <0.01* | <0.01* |
| **Age** | <0.01* | <0.01* |
| **Insurance** | <0.01* | <0.01* |
| **Language Spoken** | <0.01* | <0.01* |
| **Sex** | <0.01* | <0.01* |
| **ED Use** | >0.01 | >0.01 |
| x^2^ and fisher's exact test analysis between Isolated Rural and PEH sub-population demographics. | | |
| *Significance set at 0.05. | | |
| PEH = Patients Experiencing Houselessness | | |
