# Supplementary material for: Mobile health clinics in a rural setting: a cost analysis and time motion study of La Clínica in Oregon, United States
Source: BMC Health Serv Res. 2025 Jan 17;25:97. doi: 10.1186/s12913-024-12203-5 (PMC11740325; doi:10.1186/s12913-024-12203-5)
Supplement: Supplementary file 2 — Supplementary Material 2. Healthcare Utilization of Patient Sub-Populations. Description: Contains the methods and results breakdown of the patient procedure utilization of mobile health clinic services over the study time period. Includes breakdown and descriptive statistical analysis of the most frequent services provided by the mobile clinic and comparisons between the two patient sub-populations (Isolated Rural patients and patients experiencing houselessness). Supplementary table 3 presents the most common procedures utilized and their Medicaid cost information in table format. Supplementary figure 1 presents the change in use of the mobile clinic across two time periods, the first being the first year of operation, aligning with peak pandemic use and the second period being the second year of operation, noted as less dominated by the covid-19 pandemic, and colloquially referred to as “post” pandemic, with the acknowledgement that the definition is a transition term – not an absolute. Supplementary figure 2 and 3 compare the type of procedures used during covid and “post” covid by rural patients and patients experiencing houselessness, respectively. [file 12913_2024_12203_MOESM2_ESM.docx]

**Additional File 2: Healthcare Utilization of Patient Sub-Populations**

**Methods**

Patient resource utilization was assessed using CPT procedure codes from MHC patient records. The procedure codes were assigned a cost value based on the Oregon Health Plan 2023 fee-schedule.(28) The procedure codes were analyzed for frequency, overall and by sub-population. In addition, the utilization of procedures codes was separated into two-time frames by date to include the first half of utilization and second half. The frequency of utilization was assessed for both time periods to understand the shift in resource utilization from a covid roll out period to preventative services. Cost of each procedure was reported.

**Results**

A total of 232 distinct days of patient visits on the MHC were included in the analysis. Over the time frame, 1,981 patient encounters with 812 unique patients were recorded and included in the analysis. The main services of the MHC over this time frame were observed to be primary and preventative care for patients of all ages, including walk-in and appointments.

*Frequent Procedures Utilized.* Of the procedures utilized on the MHC, 31% of reported procedures were vaccinations (N = 438, 31%), 27% were Covid-19 related encounters (N = 377, 27%), 13% were outside referrals (N = 179, 13%), and 12% were point of care testing (POCT). Significant differences in utilization of these frequent procedures were found between sub-populations (x^2^, p < 0.01) illustrated in Supplementary Table 3. Vaccines procedures overall decreased (-61% change) between the initial (N = 313, 43%) and recent (N = 125, 18%) time frame, smaller changes over time in the rural patient population (initial N = 111, 34% to recent N=84, 28%) and no use for PEH (N= 0, 0%). Covid-19 related procedures overall decrease from 45% to 8% of procedures (-78% Δ overall), with differences between sub-populations.

Broken down, 12% of procedure codes during the one-year time frame was Moderna Covid-19 Vaccine (N = 162, 12%), followed by 7% of codes for Moderna Covid-19 2nd dose (N = 94, 7%), 4% for the influenza vaccine (N=54, 4%) and 3% A1c point of care testing (N = 46, 3%). There were significant differences in utilization of these procedures between patient sub-populations (x^2^, p < 0.01). A breakdown of the most frequently utilized procedure codes by population is presented in Supplementary Table 2.

There were significant differences in the use of procedures when comparing initial operation period to the recent operation period over all patients seen at MHC (x^2^, p < 0.01). Supplementary Figure 1.

**Supplementary Figure 1:** Top Procedure Codes Utilized on Rural Mobile Health Clinic, 2022 to 2023

**Legend**: Top procedure codes utilized on the rural mobile health clinic from April 1^st^, 2022, to April 1^st^, 2023. Includes procedure frequency from initial year of operation of the mobile health clinic in the community. Procedures were compared between the overall patient population and within patient sub-populations (PEH, migrant or seasonal patients, Native American patients, and isolated rural dwelling patients). The procedure codes were separated to assess use before (orange) and after (blue) the assigned date, 09-08-2022. POCT = Point of Care Testing. PEH = patients experiencing houselessness

Significant difference in procedure code utilization was found between sub-populations (x^2^, p < 0.01). For isolated rural patients, initial utilization was dominated by Moderna covid-19 vaccine (N=53, 16%) and Moderna Covid-19 vaccine 2^nd^ dose (N=28, 8%). Recent utilization was dominated by the influenza vaccine (N=45, 15%) and Moderna Covid-19 Bivalent Vaccine (N=33, 11%). Significant differences between initial and recent utilization of procedures by rural patients was found (x^2^, p < 0.01). See Supplementary Figure 2.

For PEH, initial utilization was dominated by referrals to behavioral health consultant (N=4, 7%) and complete blood count with differential (N=3, 5%). Recent utilization was dominated by chlamydia and gonorrhea testing (N=12, 6%) followed by syphilis testing (N=11, 6%). Variation between initial and recent utilization of procedures by PEH was not significant at 95% (x^2^, p = 0.06). See Supplementary Figure 3.

**Supplementary Figure 2**: Change in Rural Patients Procedure Utilization Between Two Time Periods

**Legend**: Top procedure codes utilized on the rural mobile health clinic from April 1^st^, 2022, to April 1^st^, 2023, separated into two periods. Includes procedure frequency from initial year of operation of the mobile health clinic in the community. Procedures were compared between two patient sub-populations, rural PEH and isolated rural dwelling patients. The procedure codes were separated to assess initial use (before the assigned date) and recent use (after the assigned date, 09-08-2022).

PEH = patients experiencing houselessness.

**Supplementary Figure 3**: Change in Patients Experiencing Houselessness Procedure Utilization Between Two Time Periods

**Legend**: Top procedure codes utilized on the rural mobile health clinic from April 1^st^, 2022, to April 1^st^, 2023, separated into two periods. Includes procedure frequency from initial year of operation of the mobile health clinic in the community. Procedures were compared between two patient sub-populations, PEH and isolated rural dwelling patients. The procedure codes were separated to assess initial use (before the assigned date) and recent use (after the assigned date, 09-08-2022).

PEH = patients experiencing houselessness.

| Supplementary Table 3: Top Procedure Code Utilization from MHC Patients, Overall and by Sub-Population | | | | | |
| --- | --- | --- | --- | --- | --- |
| **Procedure Name** | **Cost per Procedure**  **USD 2023** | **All Patients**  **N (%)** | **Rural Patients**  **N (%)** | **PEH**  **N (%)** | **P**  **X^2^** |
| **Overall** |  | **1398** | **636** | **243** | **<0.01** |
| Moderna Covid-19 Vaccine | 0.01 | 162 (12%) | 53 (8%) | 0 (0%) |  |
| Moderna Covid-19, 2nd Dose | 0.00 | 94 (7%) | 28 (4%) | 0 (0%) |  |
| Influenza Vaccine | 21.96 | 54 (4%) | 45 (7%) | 0 (0%) |  |
| A1c (POCT) | 15.00 | 46 (3%) | 24 (4%) | 8 (3%) |  |
| Moderna Covid-19 Vaccine Bivalent | 0.01 | 43 (3%) | 33 (5%) | 0 (0%) |  |
| Sars Antigen Test (POCT) | 125.00 | 38 (3%) | 12 (2%) | 1 (2%) |  |
| Covid-19 Related Encounter | 0.00 | 31 (2%) | 9 (1%) | 0 (0%) |  |
| X-Ray Referral | 0.00 | 28 (2%) | 10 (2%) | 7 (3%) |  |
| Visual Screening Acuity Test | 2.14 | 26 (2%) | 14 (2%) | 0 (2%) |  |
| Complete Blood Count with Differential | 5.44 | 24 (2%) | 13 (2%) | 9 (2%) |  |
| Hepatitis C Antibody | 9.99 | 23 (2%) | 9 (1%) | 10 (4%) |  |
| Ranked top eleven procedure codes utilized over one year of MHC intervention operation compared between patient sub-populations using x^2^ (p < 0.01). Cost per procedure unit cost reported using Oregon State Medicaid Fee Schedule 2023.  PEH = Patients experiencing houselessness; POCT = Point of care testing; % Δ = percent change | | | | | |
