# Supplementary material for: Mobile health clinics in a rural setting: a cost analysis and time motion study of La Clínica in Oregon, United States
Source: BMC Health Serv Res. 2025 Jan 17;25:97. doi: 10.1186/s12913-024-12203-5 (PMC11740325; doi:10.1186/s12913-024-12203-5)
Supplement: Supplementary file 3 — Supplementary Material 3. Full List of Time Motion Activities. Description: Includes list of activities for the task and analytic categories used during the time motion data collection for both the nurse practitioners and community health workers as well as their defined category for analysis. [file 12913_2024_12203_MOESM3_ESM.docx]

Additional File 3: Full List of Time Motion Activities

| **Supplementary Table 4.1:** Time and Motion Activities for Mobile Health Clinic Nurse Practitioner | | |
| --- | --- | --- |
| **Major Category** | **Minor Category** | **Analytic Grouping** |
| **Computer** |  |  |
|  | E-mail/teams/meeting | Indirect Care |
|  | Other | Miscellaneous |
|  | Writing Note | Indirect Care |
|  | Chart | Indirect Care |
|  | Writing Orders/Scripts | Indirect Care |
|  | Looking up info/data | Indirect Care |
| **Examine/Read** |  |  |
|  | Chart | Indirect Care |
|  | Mail | Administration |
|  | Other | Miscellaneous |
|  | Patient | Direct Care |
|  | Radiograph | Indirect Care |
|  | Schedule | Administration |
| **Looking For** |  |  |
|  | Colleague | Indirect Care |
|  | Lab Result | Indirect Care |
|  | Other | Miscellaneous |
|  | Patient | Indirect Care |
|  | Radiograph | Indirect Care |
|  | Supplies | Indirect Care |
| **Talking to Patient** |  |  |
|  | reading chart | Direct Care |
|  | putting orders | Direct Care |
|  | looking up info / data | Direct Care |
|  | examine patient | Direct Care |
|  | procedure | Direct Care |
|  | computer - other | Direct Care |
| **Talking** |  |  |
|  | Colleague/Staff – Direct Care | Indirect Care |
|  | Colleague/Staff – Other | Miscellaneous |
|  | Educating Patient | Direct Care |
|  | Other | Miscellaneous |
|  | Patient History | Direct Care |
|  | Patient Other | Direct Care |
|  | Patient's Family | Direct Care |
|  | Personal | Miscellaneous |
|  | Study Consent | Miscellaneous |
| **Phone** |  |  |
|  | Getting Results | Indirect Care |
|  | Other | Miscellaneous |
|  | Patient | Direct Care |
|  | Admin | Administration |
|  | Personal | Miscellaneous |
|  | Scheduling Tests | Indirect Care |
| **Procedure** |  |  |
|  | Joint Inspection | Direct Care |
|  | Ultrasound | Direct Care |
|  | Lab Test | Direct Care |
|  | Other | Direct Care |
| **Traveling** |  |  |
|  | driving to site/away from site | MHC |
|  | setting up MHC | MHC |
|  | call patient | MHC/Direct Care |
|  | computer | MHC |
|  | reading chart | MHC/Indirect Care |
|  | email | MHC/Administration |
|  | admin meeting | MHC/Administration |
|  | Waiting | MHC |
|  | Talking to colleague - indirect care | MHC/Indirect Care |
|  | Talking to colleague - miscellaneous | MHC |
|  | Other | MHC |
| **Direct** |  |  |
|  | Direct care | Direct Care |
| **Indirect** |  |  |
|  | Indirect care | Indirect Care |
| **Oops** |  |  |
|  | Can't Find Entry | Miscellaneous |
|  | Other | Miscellaneous |
| **Miscellaneous** |  |  |
|  | Eating | Miscellaneous |
|  | Idle | Miscellaneous |
|  | Other | Miscellaneous |
|  | Restroom | Miscellaneous |

| **Supplementary Table 4.2:** Time and Motion Activities for Mobile Health Clinic Community Health Worker | | |
| --- | --- | --- |
| **Major Category** | **Minor Category** | **Analytic Grouping** |
| Computer | Email/Teams | Admin |
|  | MHC Meeting | MHC |
|  | Other | Miscellaneous |
|  | Writing note | Indirect |
|  | Chart | Indirect |
|  | Checking patient in | Direct |
|  | Looking up information/data | Indirect |
|  | MHC related/other | MHC |
|  | Scheduling appt | Indirect |
|  | Patient Insurance | Direct |
|  | Talking to colleagues - direct care | Indirect |
|  | MHC tech | Indirect |
|  | Indirect patient care | Indirect |
|  | Patient SDOH needs care coordination | Indirect |
| Examine/Read | Mail | Admin |
|  | Other | Miscellaneous |
|  | MHC related/other | MHC |
|  | Schedule | Admin |
| Looking For | Colleague | Indirect |
|  | Patient | Indirect |
|  | Other | Miscellaneous |
|  | Patient Insurance | Indirect |
|  | Patient care supplies | Indirect |
|  | MHC supplies | MHC |
| Miscellaneous | Eating | Miscellaneous |
|  | Idle/waiting | Miscellaneous |
|  | Walking | Miscellaneous |
|  | Other | Miscellaneous |
| Oops | Can’t find entry | Miscellaneous |
|  | Other | Miscellaneous |
| Phone | Other | Miscellaneous |
|  | Patient | Direct |
|  | Admin | Admin |
|  | Personal | Miscellaneous |
|  | Clinic - scheduling appt | Indirect |
|  | Clinic - other indirect patient care | Indirect |
|  | Clinic - MHC | MHC |
|  | Clinic - Other | Indirect |
|  | Insurance company | Indirect |
|  | Pharmacy | Indirect |
|  | Community partner | CHW |
|  | Mechanic/delivery MHC | MHC |
|  | Examining MHC related | MHC |
| Procedure | Patient Vitals | Direct |
|  | Eye Exam | Direct |
|  | Lab test | Direct |
|  | Other | Direct |
| Talking | Educating patient | Direct |
|  | Patient other | Direct |
|  | Patient history | Direct |
|  | Patient Insurance | Direct |
|  | Patient SDOH | Direct |
|  | Patient care coordination | Direct |
|  | Colleague - medical | Indirect |
|  | Colleague - care coordination | Indirect |
|  | Colleague - MHC | MHC |
|  | Colleague - miscellaneous | Miscellaneous |
|  | Interpreting | Direct |
|  | Other | Miscellaneous |
|  | Study consent | Miscellaneous |
|  | Personal | Miscellaneous |
| Talking to colleague | Computer – Chart | Indirect |
|  | MHC Management | MHC |
|  | Pt social needs | Indirect |
| Direct Care | Direct | Direct |
| Indirect Care | Indirect patient care | Indirect |
| MHC Operations | MHC | MHC |
| Travelling | Driving to site/to clinic | MHC |
|  | Getting gas | MHC |
|  | MHC set up | MHC |
|  | MHC gathering supplies | MHC |
|  | Other | MHC |
|  | MHC maintenance | MHC |
|  | MHC dumping sewage | MHC |
|  | Driving: talking to colleague direct care | MHC/Indirect |
|  | Driving: talking to colleague - MHC related | MHC |
| Talking to patient SDOH | Food insecurity | Direct |
|  | Housing insecurity | Direct |
|  | Insurance needs | Direct |
|  | Clothing needs | Direct |
|  | Dental needs | Direct |
|  | Referrals/transportation | Direct |
|  | Other | Direct |
| Talking to patient | Computer | Direct |
|  | Chart | Direct |
|  | Checking patient in | Direct |
|  | Looking up information/data | Direct |
|  | MHC related/other | Direct |
|  | Scheduling appt | Direct |
|  | Patient Insurance | Direct |
|  | Procedure | Direct |
| Writing | Forms | Indirect |
|  | Other | Miscellaneous |

**Supplementary Table 4.3**: Community Health Worker Role Specific Categories Broken Down by Tasks

| **Analytic Group** | **Major Category: Minor Category** |
| --- | --- |
| **Administration** | |
|  | Computer: Email/Teams Meeting Admin |
|  | Computer: other |
|  | Examine/rad: schedule |
| **Care Coordination** | |
|  | Computer: scheduling appt |
|  | Computer: talking to colleagues |
|  | Phone: clinic - Other |
|  | Phone: clinic - other indirect patient care |
|  | Phone: community partner |
|  | Talking: colleague - care coordination |
|  | Talking: colleague - medical |
|  | Talking: patient care coordination |
| **Miscellaneous** | |
|  | Miscellaneous: eating |
|  | Miscellaneous: idle/waiting |
|  | Miscellaneous: walking |
|  | Phone: other |
|  | Talking: colleague: miscellaneous |
| **MHC Operations** | |
|  | Computer: meeting MHC |
|  | Computer: MHC related/other |
|  | Examine/read: MHC related/other |
|  | Looking for: supplies MHC |
|  | MHC Operations |
|  | Phone: clinic - MHC |
|  | Phone: mechanic/delivery MHC |
|  | Talking to colleague: MHC management |
|  | Talking: colleague - MHC |
|  | Talking: patient care coordination |
|  | Traveling: driving to site/driving to clinic |
|  | Traveling: driving: talking to colleagues - Direct care |
|  | Traveling: driving: talking to colleagues - MHC |
|  | Traveling: dumping sewage MHC |
|  | Traveling: getting gas |
|  | Traveling: MHC maintenance |
|  | Traveling: MHC set up |
|  | Traveling: other |
| **Patient Medical Care** | |
|  | Computer: chart |
|  | Computer: checking patient in |
|  | Computer: patient indirect care |
|  | Direct Patient Care |
|  | Indirect Patient Care |
|  | Looking for: patient |
|  | Looking for: supplies patient care |
|  | Phone: Patient |
|  | Talking to patient: checking patient in |
|  | Talking to patient: computer |
|  | Talking to patient: looking up information/data |
|  | Talking: educating patient |
|  | Talking: patient history |
|  | Talking: patient other |
| **Social Determinants of Health (SDOH)** | |
|  | Computer - Patient SDOH needs coordination |
|  | Computer: patient insurance |
|  | Phone: insurance company |
|  | Phone: pharmacy |
|  | Talking to colleague - pt social needs |
|  | Talking to patient SDOH: housing insecurity |
|  | Talking to patient SDOH: insurance needs |
|  | Talking to patient SDOH: other |
|  | Talking to patient SDOH: referrals/transportation needs |
