# Supplementary figures and images for: Mobile health clinics in a rural setting: a cost analysis and time motion study of La Clínica in Oregon, United States
Source: BMC Health Serv Res. 2025 Jan 17;25:97. doi: 10.1186/s12913-024-12203-5 (PMC11740325; doi:10.1186/s12913-024-12203-5)

## Top CPT Codes, All Patients

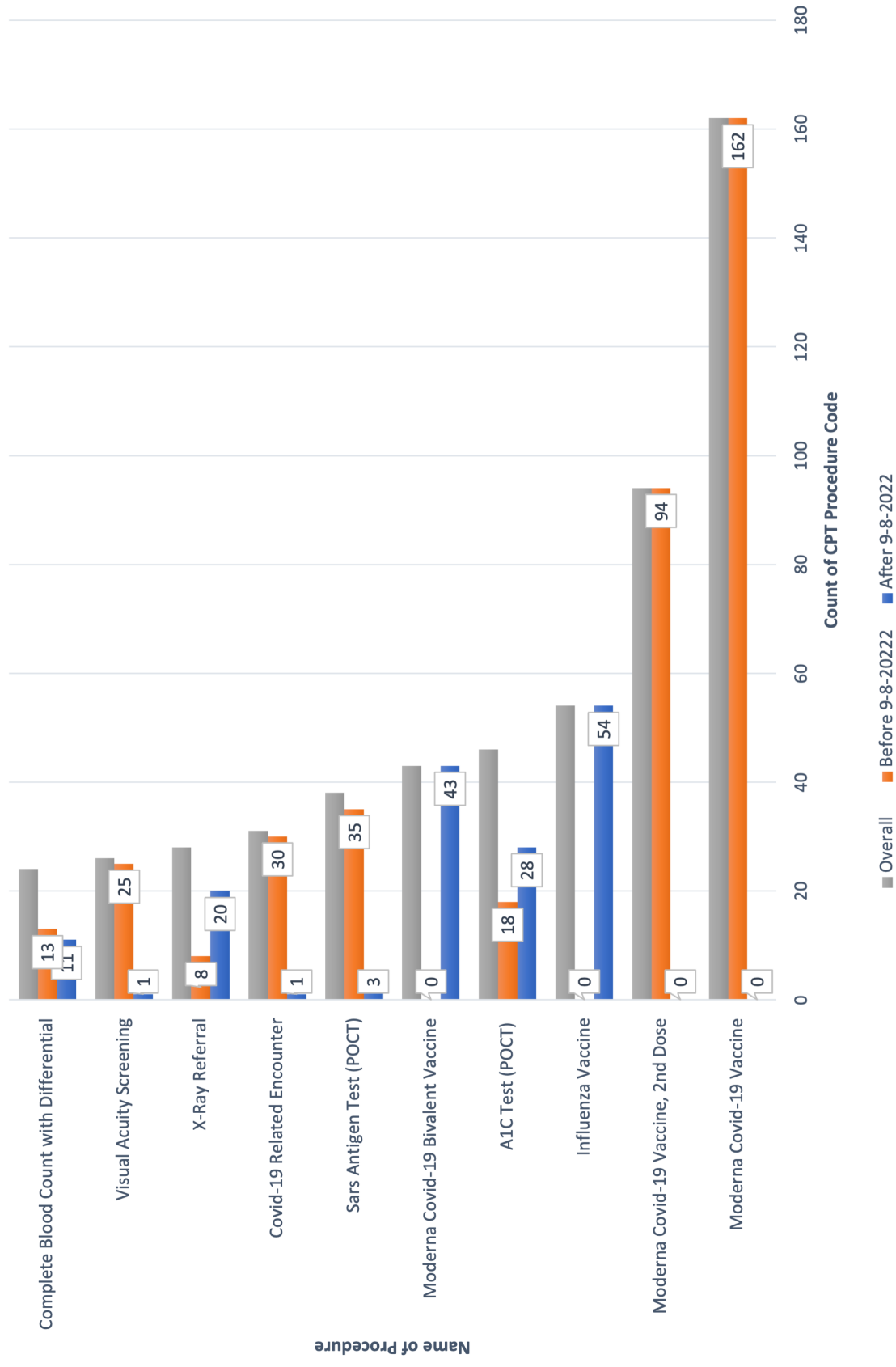

Supplement: Supplementary file 4 — Supplementary Material 4. Supplementary Figure 1: Top procedure codes utilized on Rural mobile health clinic, 2022 to 2023. [file 12913_2024_12203_MOESM4_ESM.pdf]

Top 10 Mobile Medical Unit Procedures Utilized

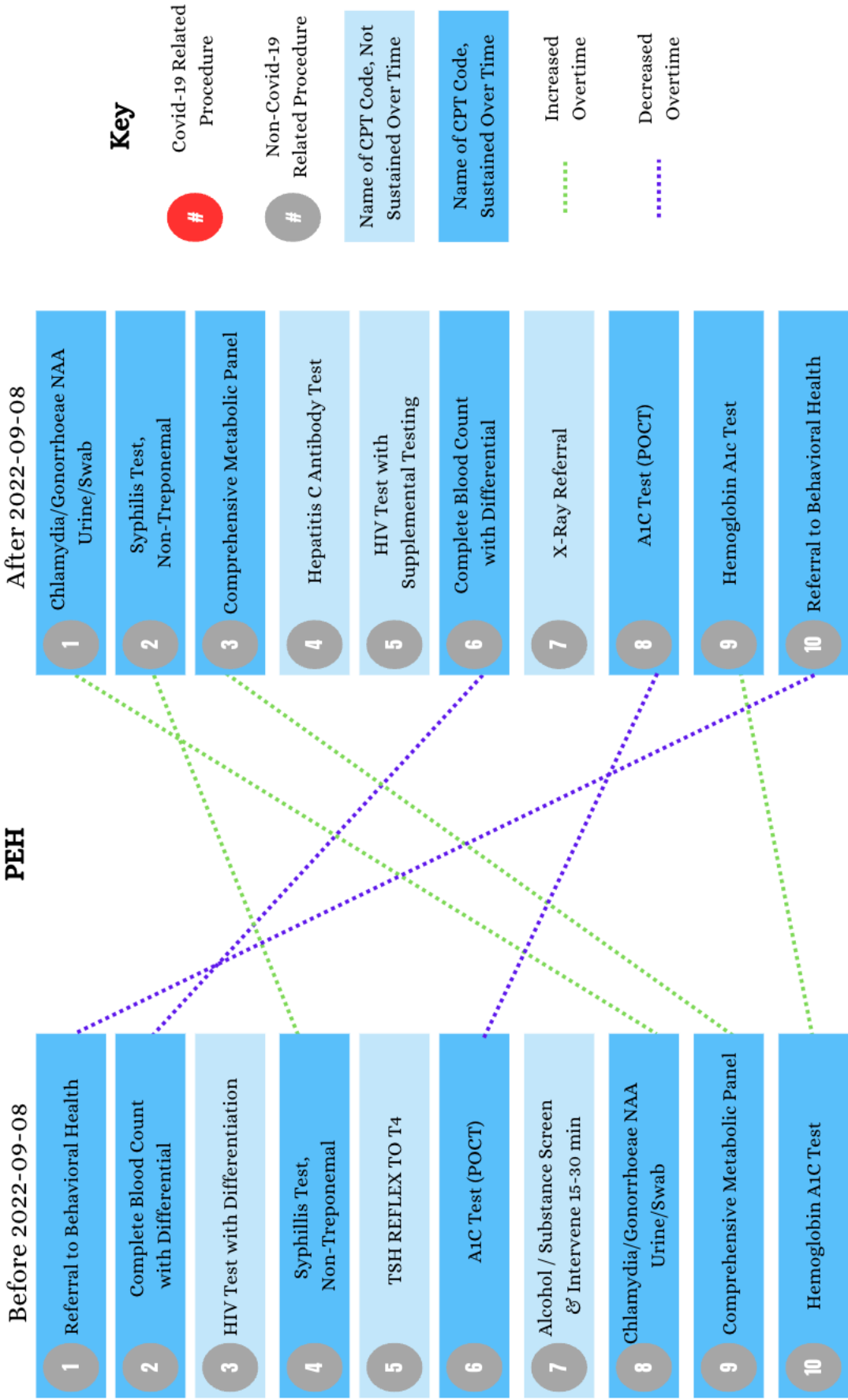

Supplement: Supplementary file 6 — Supplementary Material 6. Supplementary Figure 3 Change in patients experiencing houselessness procedure utilization between two time periods. [file 12913_2024_12203_MOESM6_ESM.pdf]
