# Supplementary material for: Mobile health clinics in a rural setting: a cost analysis and time motion study of La Clínica in Oregon, United States
Source: BMC Health Serv Res. 2025 Jan 17;25:97. doi: 10.1186/s12913-024-12203-5 (PMC11740325; doi:10.1186/s12913-024-12203-5)

Top 10 Mobile Medical Unit Procedures Utilized
Rural Patients

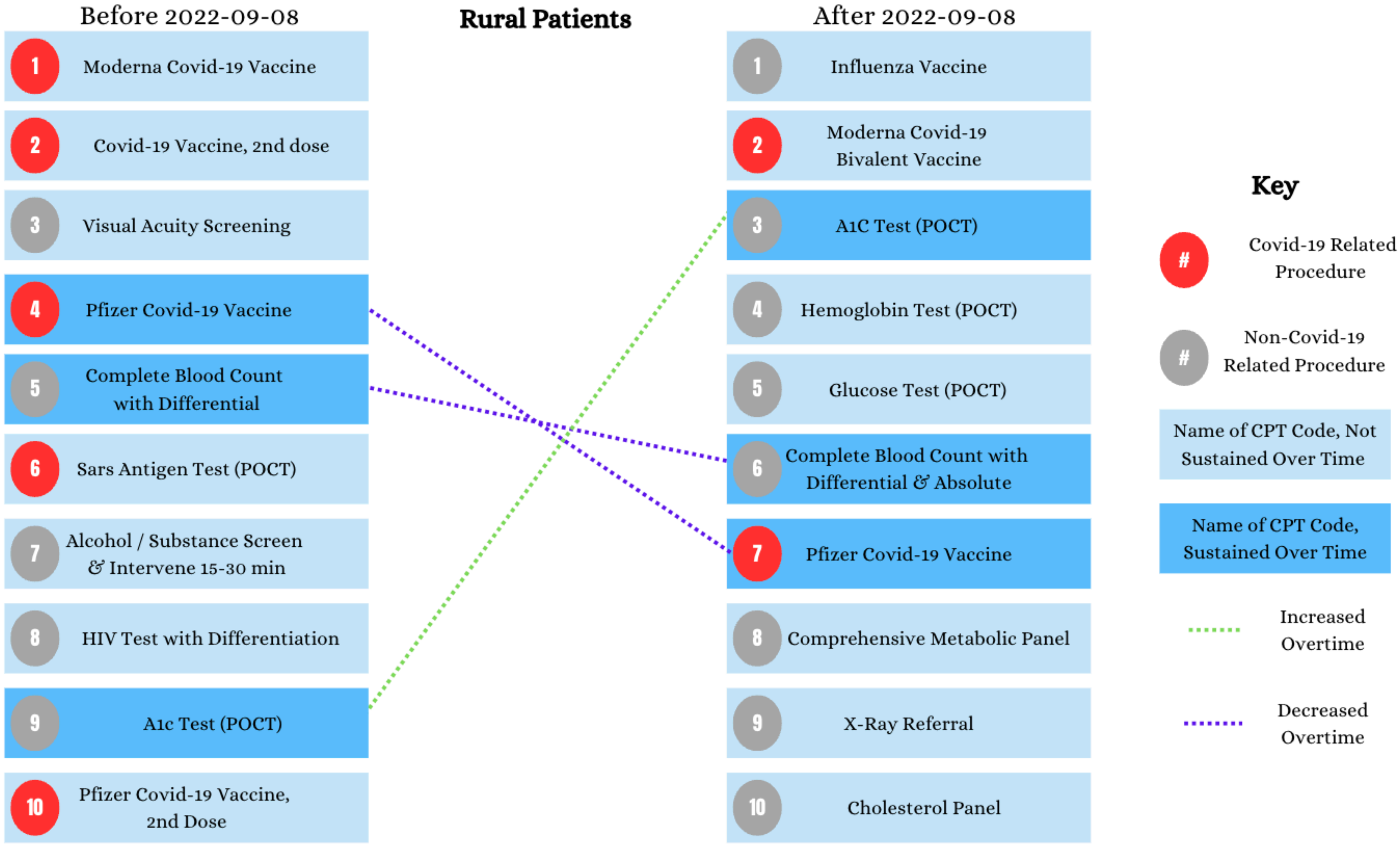

Supplement: Supplementary file 5 — Supplementary Material 5. Supplementary Figure 2: Change in Rural patients procedure utilization between two time periods. [file 12913_2024_12203_MOESM5_ESM.pdf]
